# Development and implementation of a Telenephrology dashboard for active surveillance of kidney disease: a quality improvement project

**DOI:** 10.1186/s12882-020-02077-0

**Published:** 2020-10-06

**Authors:** Melissa L. Swee, M. Lee Sanders, Kantima Phisitkul, George Bailey, Angie Thumann, Nikki Neuzil, Bharat Kumar, Amy M. J. O’Shea, Bradley S. Dixon

**Affiliations:** 1 Division of Nephrology, University of Iowa Hospitals and Clinics, 200 Hawkins Drive, Iowa City, IA 52245 USA; 2 Iowa City Veterans Affairs Health Care System, Iowa City, USA; 3 Division of Immunology, University of Iowa Hospitals and Clinics, Iowa City, USA; 4 Division of Internal Medicine, University of Iowa Hospitals and Clinics, Iowa City, USA

**Keywords:** Kidney diseases, Quality improvement, Telemedicine, Rural health

## Abstract

**Background:**

Kidney disease accounts for more than 49 billion dollars in healthcare expenditures annually. Early detection and intervention may reduce the burden of disease. We describe a quality improvement project to develop a telenephrology dashboard that proactively monitors kidney disease.

**Methods:**

One hundred eighty-four thousands Veterans within the Iowa City Veterans Affairs Health Care System were eligible for telenephrology consultation. The dashboard accessed the charts of 53,085 Veterans at risk for kidney disease. We utilized Lean-Six Sigma tools and principles and the Define-Measure-Analyze-Improve-Control Framework to develop and deploy a telenephrology dashboard in 4 community-based outpatient clinics (CBOCs). The primary measure was the number of days to complete consultation. Secondary measures included number of electronic consultations per month, distance and cost of Veteran travel saved, and number of steps for completion of consult.

**Results:**

The data of 1384 Veterans at the 4 CBOCs were analyzed by the telenephrology dashboard, of which 459 generated telenephrology consults. The number of days to complete any type of consultation was unchanged (48.9 days in 2019, compared to 41.6 days in 2017). The average Veteran saved between $21.60 to $63.90 per trip to Iowa City. Between March 2019 and August 2019, there were 27.3 telenephrology consults per month. The number of steps needed to complete the consult request was decreased from 13 to 9.

**Conclusions:**

Utilization of the telenephrology dashboard system contributed to an increase in consultations completed through electronic means without decreasing face-to-face consults. Electronic consults now outnumber traditional face-to-face consultations at our institution. Telenephrology consultation improved early detection and identification of kidney disease and saved time and costs for Veterans in travel, but did not decrease the average number of days to complete consultation requests.

## Background

Kidney disease is a major contributor to disease burden in the United States, affecting 30 million individuals [1], and accounting for more than 49 billion dollars per year in healthcare costs in the Medicare population alone [2]. Early detection of at-risk patients in primary care clinics and timely consultation with kidney specialists may potentially reduce this burden [3]. However, the lack of efficient coordination between primary care and nephrology clinics often leads to delays in care and missed opportunities for early intervention [4]. The global nephrology workforce shortage further exacerbates the problem and highlights the need to develop newer tools to more efficiently evaluate and care for patients with early signs of kidney disease [5].

The development and utilization of dashboards may help to address this problem. In recent years, dashboards have been developed in several settings to pro-actively identify and manage conditions, including kidney disease [6]. Studies suggest that dashboards may help to improve adherence to quality guidelines and improve patient outcomes [7]. Because the practice of nephrology is largely dependent upon monitoring of laboratory results and vital signs, development of a dashboard for kidney diseases is an appealing quality improvement approach [8].

The Iowa City Veterans Affairs (VA) Health Care System is a member of the Veterans Affairs Midwest Health Care Network that serves more than 184,000 Veterans living in 50 counties spread throughout the mostly rural areas of Eastern Iowa, Western Illinois, and Northern Missouri. While the hospital and specialized clinics, including Nephrology, are headquartered in Iowa City, there are also 10 community-based outpatient clinics (CBOCs) in Iowa and Illinois that deliver primary care [9]. When specialist services are required, patients are either referred to the clinics in Iowa City or receive care in the community outside of the VA system. This often poses problems for coordination of care between primary care practitioners and nephrologists. This quality improvement initiative aims to facilitate such coordination through the development of a dashboard.

## Methods

This study was submitted to the Institutional Review Board at the Iowa City Veterans Affairs Health Care System. As a Quality Improvement initiative, it was exempt from full review.

We utilized Lean Six Sigma principles and tools as well as the Define-Measure-Analyze-Improve-Control (DMAIC) Framework to structure our intervention (Table 1). Lean Six Sigma is a well-validated set of methods to systematically engage in quality improvement, and the DMAIC Framework has been utilized in multiple healthcare settings to effect successful changes in practice [10, 11].

**Table 1:** DMAIC (Define-Measure-Analyze-Improve-Control) Framework

	Element of each step	Actions Performed by Core Investigators
**Define**	Problem statement	Project Charter was drafted, including a problem statement that articulated the background, specific needs, and aims of the project
	Goal statement	Articulated in the Charter: “Decrease number of days between the PCP entering an order for any type of nephrology consult and the nephrologist completing a consult note from 48.9 days to 30 days, by August 2019.”
	Project scope	Articulated in the Charter: “This Lean Six Sigma project will take place 360 days from start to validated solutions”
	Identification of project sponsor	The Chief of Medical Services was identified and designed as the project sponsor
	Identification of process owner	The telenephrology Case Manager was identified and designated as the process owner
	Primary impact measure	Articulated in the Charter: “Number of days between primary care practitioner entering a consult and nephrologist performing chart review”
	Secondary impact measures	Travel time and costs saved by Veterans Number of telenephrology consults generated
**Measure**	Baseline operations	Chart review determined baseline number of days to completion of nephrology consultation Pre-Intervention Process Map
	Impact data	Project charter updated regularly based on performance
**Analyze**	Process mapping	Current process map from PCP entry of lab order to completion of consult order is shown in Fig. 2
	Voice of the customer	Veterans, PCPs, and Nephrology clinical staff (practitioners and case managers) interviewed Point-of-care observations performed
**Improve**	Future state maps	Future state map from PCP entry of lab order to completion of consult order is shown in Fig. 2
	Plan-Do-Study-Act cycles	Five PDSA cycles were performed
	Pilot results	Gaps between predicted and actual performance analyzed
**Control**	Development of a control plan	A control plan was drafted to summarize the process and take steps to ensure that the level of improvement is maintained and sustained
	Impact summary	A one-page impact summary was drafted with simplified language and diagrams for dissemination within the institution
	Recognition of work accomplished	Results of quality improvement project disseminated locally at institutional grand rounds and nationally at annual conferences Celebration of short-term wins

The Quality Improvement Team used the DMAIC Framework to guide the intervention

### Study sites and participants

This initiative took place at 4 CBOCs within the state of Iowa: Ottumwa, Quincy, Galesburg, and Dubuque. These 4 CBOC’s have 23 primary care practitioners (PCPs), of which 9 participated in the quality improvement project. These 9 PCPs serve approximately 9000 patients, of which 93% live in rural or very rural areas. Like in most VA settings, the majority of patients are male (in this case, 97.4%). The telenephrology nurse coordinator screened patients to determine eligibility for telenephrology consultation, based on whether they had chronic kidney disease Stage 3b or above (eGFR < 45), acute kidney injury, hematuria, or proteinuria. Patients who were already seeing a non-VA nephrologist were excluded, and those that were seeing a VA nephrologist were screened to determine if there was a new complication that warranted a telenephrology consultation.

### Defining and measuring the problem

At the start of this initiative, we drafted a project charter outlining the aims, scope, participants, outcomes, and benchmarks for the project. A multidisciplinary core team of two nephrologists (KP and MLS), two PCPs (DC and MH), a quality improvement scholar (MS), two nephrology nurse coordinators (AT and NN), one PCP nurse coordinator (TB), a health informatics expert (GB), and the chief of medical service (BSD) was assembled. PCPs and nephrologists were consulted at every step of drafting the project charter and developing protocols.

The primary impact measure was the number of days between the PCP entering an order for any type of nephrology consult and the nephrologist completing a consult note. In addition to traditional face-to-face encounters, we also included e-consults, where PCPs enter a consult order and nephrologists review the order without contacting the patient, and Clinical Video Telehealth (CVT) appointments, where encounters between nephrologists and patients are conducted through video conferencing technology. Administrative data in the three months prior to the initiation of the project were used to determine the baseline, i.e. status of clinic operations prior to the intervention.

**Fig. 1:**
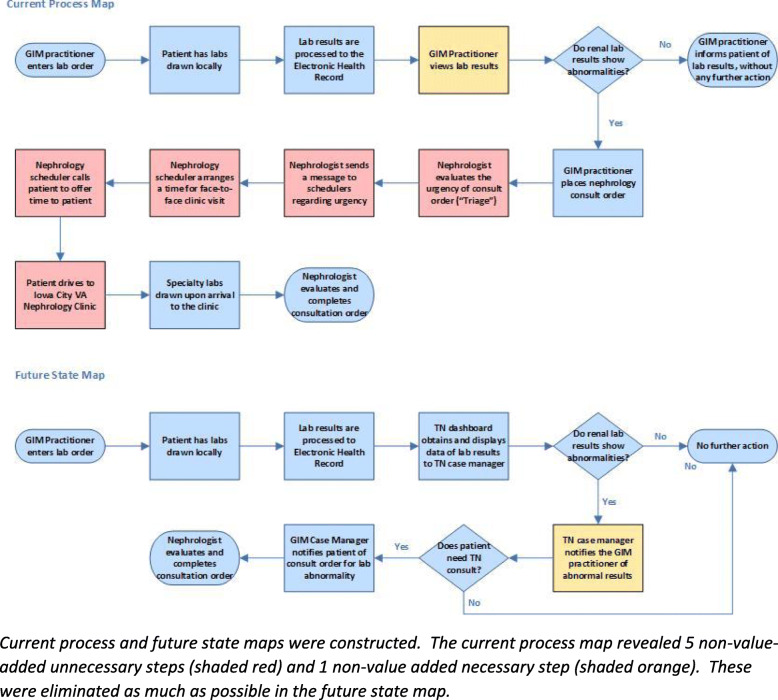
Current Process Map & Future State Map

Secondary measures included the number of telenephrology consults and the amount and cost of travel per Veteran. These were obtained through administrative data and the electronic health record. Baseline operations were also documented through the creation of a process map (Fig. 1). MS, AT, and NN followed and documented the individual steps by which a patient is referred to nephrology clinic. This process map depicts the way in which patients with kidney disease come into contact with nephrologists.

### Analysis of data

We obtained data from three major sources: [1] the electronic health record, [2] administrative data, and [3] transcripts of group interviews.

The electronic health record was used to obtain patient data, including serum creatinine, estimated glomerular filtration rate (eGFR), urine blood, urine protein-to-creatinine ratio, urine microalbumin, and systolic blood pressure (SBP).

Administrative data were collected for certain outcomes, such as the number of days to complete the consult, demographic profiles of patients (location, age, sex, and stated ethnicity) and number of new face-to-face, electronic, and telenephrology consults.

Lastly, we conducted interviews with PCPs, nephrologists, case managers, and Veterans, which were transcribed and analyzed by MS and AT.

### Voice of the customer

The investigative team identified a set of four key stakeholders: PCPs, nephrologists, clinic case managers, and Veterans. Throughout each step of the process, we consulted with the four key stakeholders. Three open-ended questions were asked at each of the interviews: [1] what are your overall thoughts about the current status of the program? [2] how they would change the program? And [3] is the program improving care for Veterans? Group interviews, conducted by BD and AT via video conferencing, with PCPs (*n* = 9), nephrologists (*n* = 4), and clinical case managers (n = 9) revealed three themes: [1] delay in getting patients to clinic, [2] concerns about increased workload, and [3] concerns about losing autonomy.

With regards to Veterans, individual interviews were conducted by AT. Veterans voiced frustration at delays in coordinating care and in travel to Iowa City for specialty consultation.

### Process mapping

Based on these insights, the investigative team examined the process map to identify areas for improvement and drafted a future state map (Fig. 1). Of the 13 steps leading to one of two outcomes, there were 6 steps that were deemed to be non-value added, and only 1 non-value-added step was deemed necessary. This enabled the team to focus on developing approaches and solutions that would reduce or eliminate these steps altogether, and therefore, streamline nephrology care into PCP clinics.

### Improvement and refinement of processes

#### Future state maps

These efforts culminated in the development of a future state map composed of 9 steps leading to one of two outcomes: consultation or no consultation. All six non-value-added steps from the current state map were eliminated, but one non-value-added step was introduced in the future state map.

#### Plan-do-study-act cycles

**Fig. 2:**
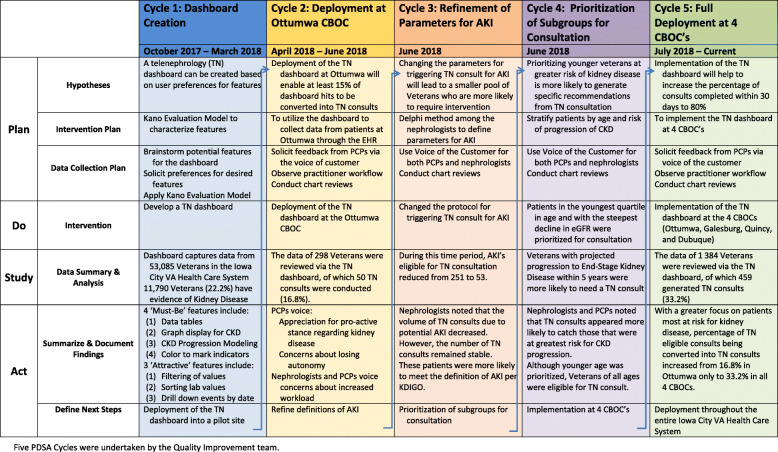
Plan-Do-Study-Act Cycles

We structured our improvement phase into five Plan-Do-Study-Act (PDSA) cycles: [1] dashboard design and development, [2] deployment at the Ottumwa CBOC, [3] refinement of parameters for Acute Kidney Injury (AKI), [4] prioritization of subgroups for consultation, and [5] full deployment at the 4 CBOC’s (Fig. 2).

##### Cycle 1: dashboard design and development

The dashboard was developed using an iterative process that cycled through [1] collection of user requirements, [2] development of dashboard prototypes, and [3] evaluation of the prototypes. First, the six individuals who would use the dashboard (MS, MLS, KP, AT, GB, and BD) were assembled to collectively brainstorm potential dashboard features, leading to a list of 8 proposed features.

**Fig. 3:**
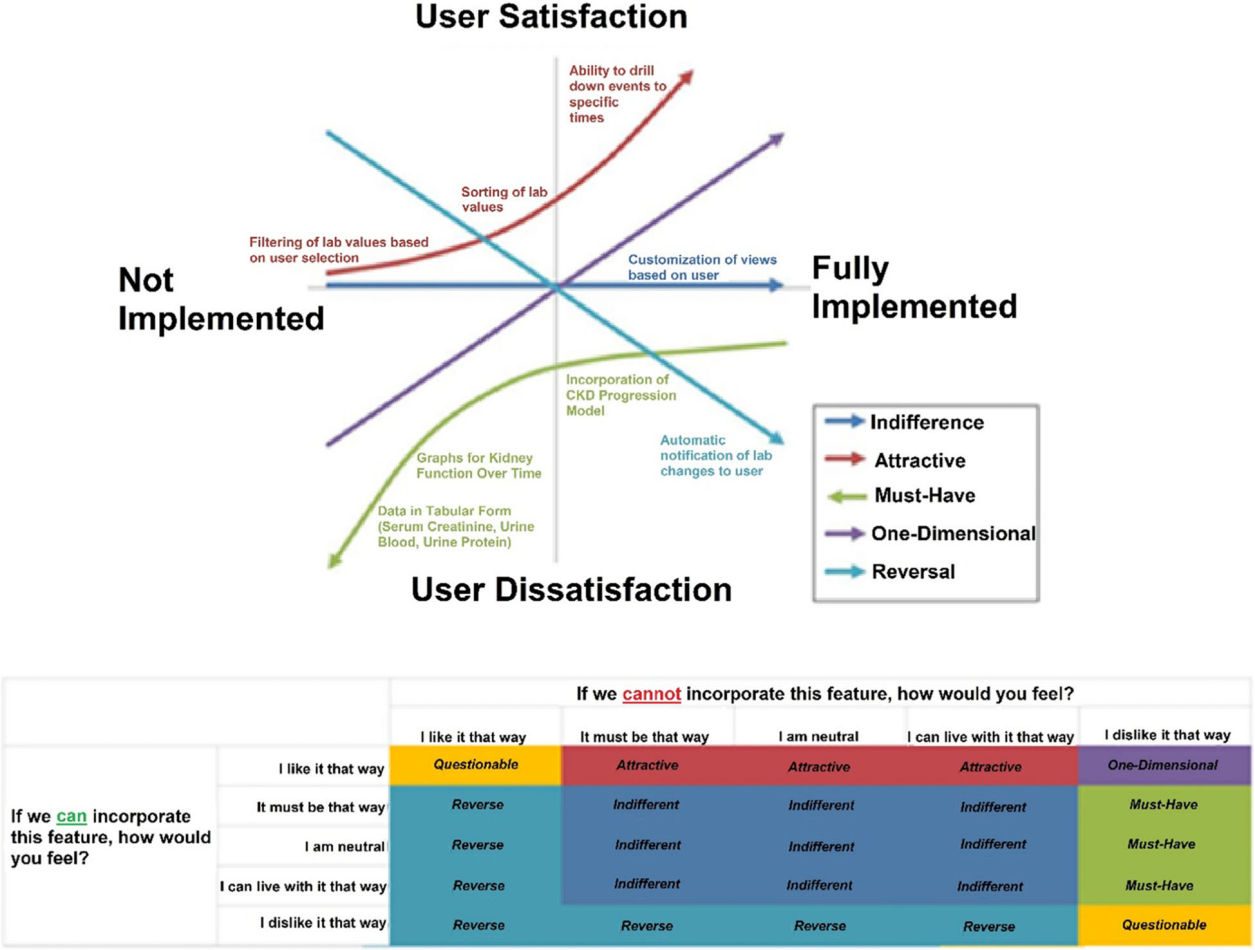
Kano Analysis for Dashboard Development

To better evaluate priorities for development and evaluation, the quality improvement team used the Kano evaluation model to better categorize features as ‘Must-Have,’ ‘Attractive,’ ‘Indifferent,’ ‘Questionable,’ ‘Reverse,’ or ‘One-Dimensional’ (Fig. 3) [12]. Due to the small sample size of users and high degree of agreement, further calculation of satisfaction and dissatisfaction coefficients were not deemed necessary.

‘Must-Have’ features included data tables for displaying serum creatinine, urine blood, and urine protein, graphs for kidney function over time, incorporation of prediction models for development of CKD, and use of color to mark indicators. ‘Attractive’ features included filtering of values based on user selection, sorting lab values and ability to drill down to specific events in time. One ‘Indifferent’ feature was identified: customization of views based on user, and one ‘Reverse’ feature was noted: automatic notifications of lab changes to users.

The ‘Must-Have’ and ‘Attractive’ features were incorporated into draft versions of the dashboard. Incorporation of the ‘Indifferent’ feature was reserved as an option for the future but there was no demand for its subsequent incorporation.

##### Cycle 2: implementation – pilot site

We piloted the dashboard at the Ottumwa CBOC, the most rural of the CBOCs (97.3%), for 3 months. We utilized point-of-care observations to ensure that the dashboard was working effectively and without technical problems. After 12 weeks, we reviewed the data and conducted group interviews with the nephrologists, PCPs, and case managers to identify areas of further improvement. The large volume of telenephrology requests suggested a need to refine parameters for AKI and drove the next PDSA cycle.

##### Cycle 3: refinement of parameters for AKI

Because 16.8% of dashboard hits resulted in telenephrology consults, the nephrologists and PCPs were concerned that the dashboard was too sensitive in picking up changes in eGFR. Therefore, the quality improvement team used the Delphi method to refine the parameters for AKI, from values of predicted eGFR below the confidence interval for the 95th percentile to a 70% prediction interval. This reduced the number of dashboard notifications for AKI from 251 to 53.

##### Cycle 4: prioritization of subgroups for Telenephrology consultation

Similarly, due to the high volume of dashboard hits, the quality improvement team sought to prioritize those who would theoretically obtain the greatest benefit from early telenephrology consultation. Veterans with projected progression to end-stage kidney disease within 5 years, and those whose age < 85 were prioritized, although all patients, regardless of age and risk of end-stage kidney disease were evaluated for possible telenephrology consultation. This change in workflow did not objectively change the number of eligible consults but rather facilitated triage of consults when volume was high.

##### Cycle 5: implementation at multiple sites

At month 10, the team began to sequentially expand the number of sites from 1 to 4, while continuing to collect data on the primary and secondary outcomes. A major concern was increasing the yield of the dashboard so that there was appropriate sensitivity of the telenephrology dashboard for generating telenephrology consults. The addition of new PCPs at other sites also provided opportunities to explore how the program can expand the scope to all 10 CBOCs within the Iowa City VA Health Care System.

### Control plan

In order to build on these successes, the team composed a control and sustainability plan. This control and sustainability plan outlined how the project would continue to evolve beyond the initial 12 months. This included celebration of the short-term wins and dissemination of our data. Additionally, we derived protocols for sustaining telenephrology consultations for nephrologists and case managers (Supplement).

## Results

### Dashboard development

**Fig. 4:**
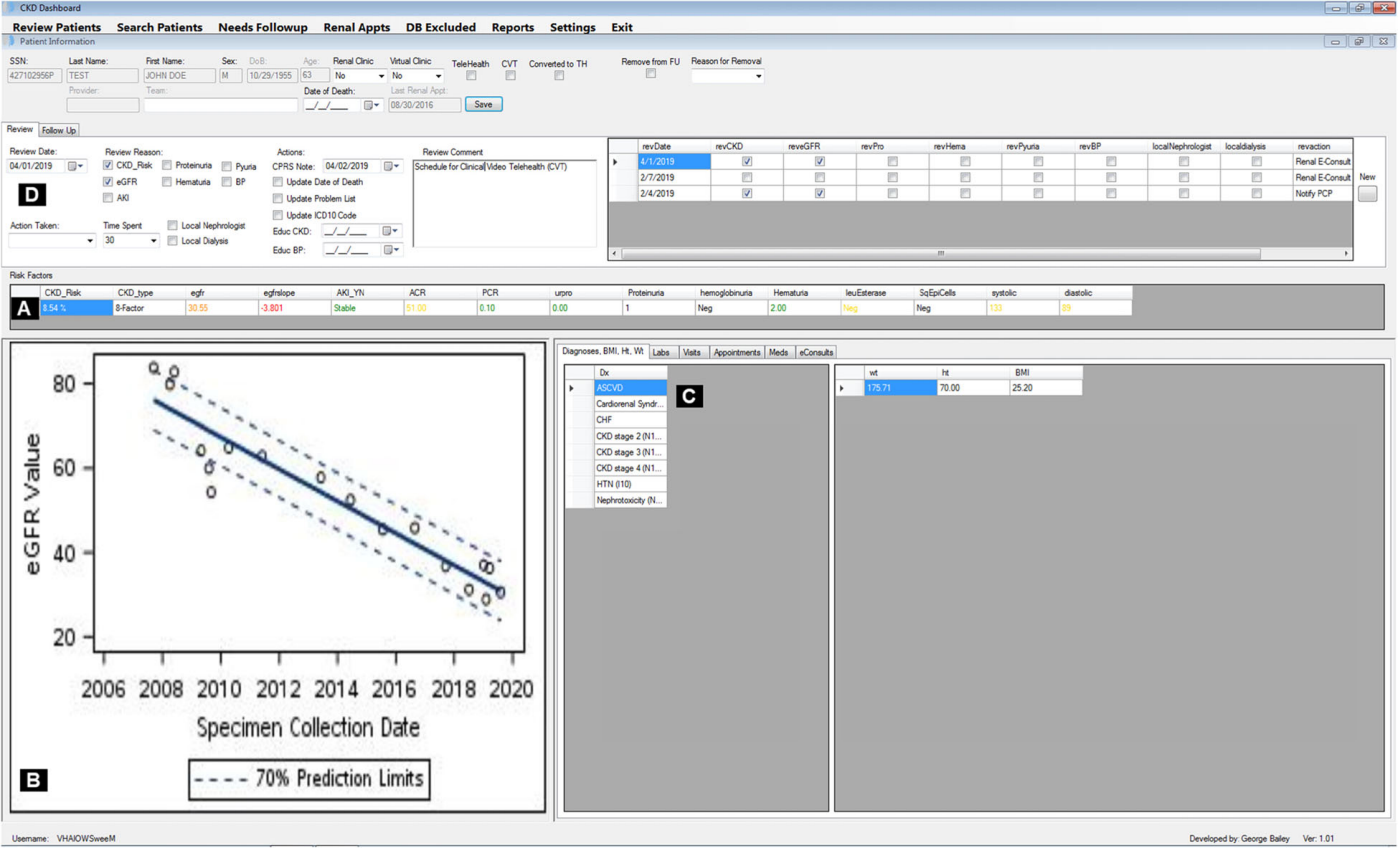
Dashboard Snapshot

Based on the results of the Kano Analysis, GB developed the telenephrology dashboard software. The ‘Attractive’ and ‘Must-Have’ features were incorporated, as shown in Fig. 4. After two iterations, the dashboard was finalized and ready for use on March 2018. Since that time, the dashboard has been accessed daily, principally by the telenephrology case manager (AT).

### Process measures

We identified 6 steps that were non-value-added, of which only 1 was necessary. Based on these, we constructed a future state map and worked towards its realization.

The future state map reduces the number of steps for completion of consultation from 13 to 9. Notable steps that were eliminated included triage by a nephrologist, scheduling the patient, and Veteran travel to the Iowa City nephrology clinic.

### Primary outcome

Throughout the project, we continued to look at the number of days to completing the consultation request for all face-to-face, electronic, and telenephrology consult orders. There was a change in average time from 41.6 days in 2017 to 28.6 days in 2018 and 48.9 days in 2019 (to July 2019), but these changes were not statistically significant (*p* = 0.57).

### Secondary outcomes

#### Veteran travel time and cost

We saved 47,718 miles of travel for the 459 Veterans in total (mean: 108 miles; median: 100 miles). This amounted to $21.60 to $63.90 saved per Veteran, depending on the formula used by the Internal Revenue Service to calculate standardized miles.

#### Telenephrology consultations

**Fig. 5:**
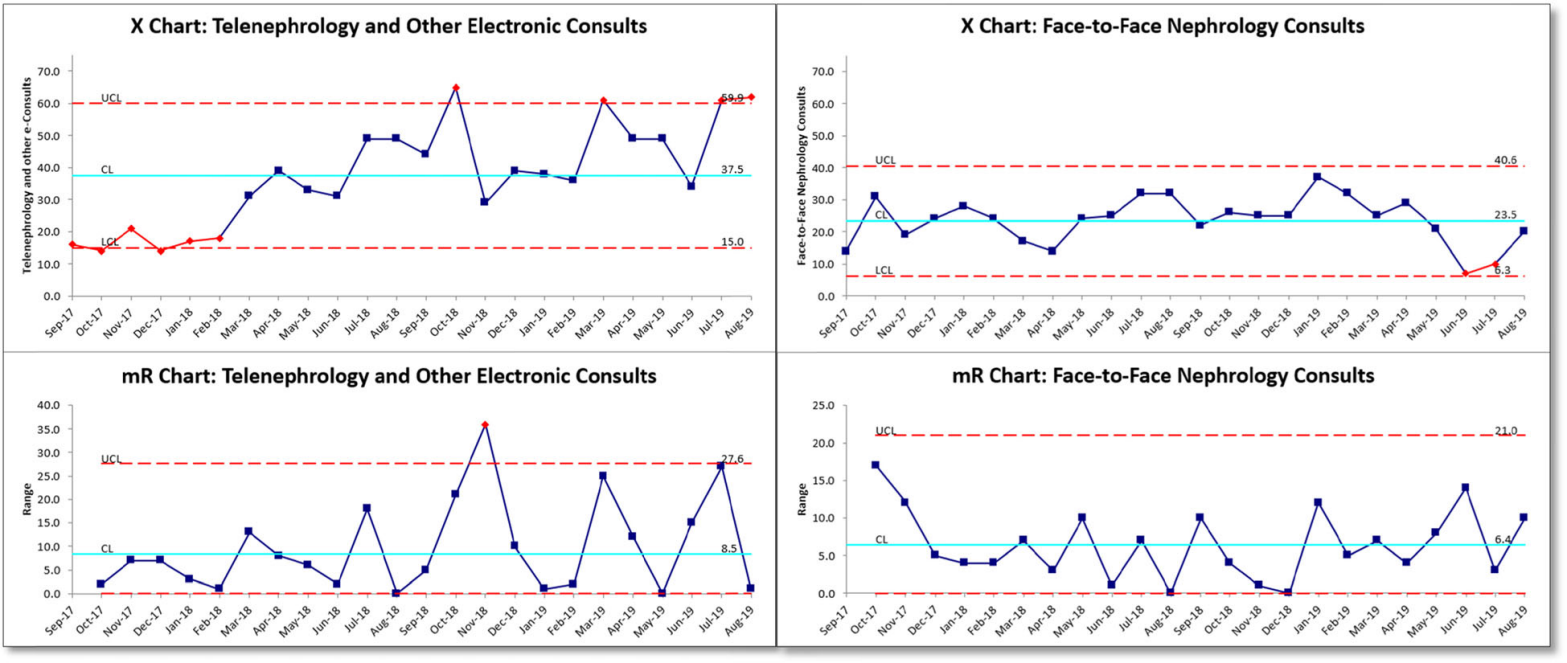
Run Chart (XmR) of Nephrology Consults

We tracked the numbers of telenephrology consults per month over the course of the 21 months. As Fig. 5 illustrates, the number of telenephrology and other electronic consultation methods has consistently outpaced traditional face-to-face consultation since March 2018, when the team started to utilize the dashboard. Since that time, the system has been largely in control with special cause variation in four of these months. In all of these cases, the number of such electronic consultations has actually exceeded the upper control limit. As of July 2019, the majority of these electronic consultations are now telenephrology consults (55.7%). Over the last six months of the project (March to August 2019), the average number of consults was 27.3 per month.

## Discussion

Multiple lines of evidence suggest that early detection and management of kidney disease can help to guide more appropriate preventative care, prevent the onset of end-stage kidney disease, and possibly lower healthcare costs [13, 14]. This quality improvement project helps to address this challenge through the creation of a dashboard for telenephrology consultation. Our experience has important implications for both PCPs and nephrologists as healthcare systems are pressed to improve value and minimizing waste.

First and foremost, we demonstrated how complex healthcare systems can directly integrate nephrologist care into primary care clinics in a way that is dynamic, synergistic, and responsive. There have been trends in the modern American healthcare system towards increasing specialization and separation of nephrologists from primary care. This separation poses a set of challenges for patients that may lead to delays in obtaining care [15, 16]. The collaborative process between nephrologists and PCPs that led to the creation of this dashboard is an example of how technology and quality improvement methodologies can be employed to offset the adverse effects of increasing specialization.

Secondly, we have developed a simple, scalable, and flexible dashboard system to monitor kidney disease among patients receiving primary care in the Iowa City Veterans Affairs healthcare system. Dashboards have long been promoted as a means to monitor conditions and diseases, but the quality of a dashboard depends largely upon usability. By using a systematic method to evaluate user needs and desires, we have created a program that is more likely to be integrated into the clinical environment and associated workflows. As the program grows, this systematic method can be employed once again for its redesign.

Thirdly, we have developed software to engage in proactive surveillance of kidney health. The quality improvement team discovered that the dashboard was initially too sensitive for acute kidney injury to be a meaningful instrument for early detection and intervention. Rather, it required refinement of parameters in order to prioritize those who would gain the most benefit from early detection, monitoring, and intervention.

### Lessons learned for other institutions

We have been able to successfully implement this telenephrology consult system using the dashboard tool described above. Our investigative team has gleaned several insights from our experience that may be informative for other institutions aiming to develop similar dashboards and telenephrology consult systems.

Most importantly, it is vital to develop a multidisciplinary team that represents the interests of various stakeholders. In our case, we ensured that nephrologists and PCPs were incorporated as core team members. Similarly, we relied upon the expertise of nephrology and primary care case managers to understand the less visible back-office processes that govern consultation behavior. The involvement of the Chief of Medical Services as the project sponsor, was instrumental in marshalling the resources, time, and talent to ensure the success of the program.

Secondly, projects of this scale demand close coordination and proactive planning. The drafting and ratification of a project charter enabled team members to better understand their positions and responsibilities. Benchmarks for dashboard development and implementation permitted the team to remain committed to the task at hand. Beyond our team members, we coordinated closely with the individual clinics, advertising the availability of this service and sought feedback from PCPs at the forefront.

Thirdly, we utilized the Kano Model to understand users’ needs with respect to dashboard design. The Kano Model is a systematic manner in which features can be categorized and prioritized for development. While our nephrologists prioritized certain features on their individual experiences, interests, and concerns, specialists from other fields may have other priorities. Therefore, using the Kano Model may help to bolster the flexibility of the intervention.

### Limitations

Despite the potential of this dashboard system to engage in active surveillance of kidney disease in PCP clinics, there remain some important limitations.

First, it is unclear how generalizable and sustainable our findings are, since our project was implemented in only four clinics in the states of Iowa and Illinois. These clinics are predominantly rural in nature. As with all quality improvement projects, it is important to replicate findings in other settings, and we are currently pursuing this approach throughout the Iowa City Veterans Affairs Health Care System.

Secondly, our primary outcome of reducing the time to complete consultation was not met. The high variability is a major reason why we could not detect a change; additionally, the volume of new patients identified by the telenephrology dashboard system likely mitigated reductions in the time to complete consult requests. Yet there were other benefits of the telenephrology consult system, including improved workflow, early detection of certain kidney diseases, and savings in cost and time for Veterans. Therefore, even if consultations were not completed more rapidly, the intervention helped to reduce unwarranted waste for patients and practitioners.

Thirdly, the overarching larger impact on kidney health, i.e. progression to end-stage renal disease, has not yet been studied. We intend on continuing this project and collecting data to understand how better integration of nephrology into PCP clinics through the dashboard impacts the progression of chronic kidney disease and proteinuria over longer periods of time.

## Conclusions

We were able to develop a system of actively monitoring kidney disease through a telenephrology dashboard system, and deploy it in 4 predominantly rural community-based outpatient clinics. Although it did not result in decreased time to complete consultation, it enabled a larger volume of Veterans to receive specialist expertise within the context of their PCP clinics. The majority of nephrology consultations at our center are now completed electronically, and the majority of these consults are telenephrology consults. Veterans saved an average of $20.60 to $63.90 per trip to the nephrology clinic. The quality improvement team continues to adapt this intervention for wider scale implementation in all 10 community-based outpatient clinics in the Iowa City Veterans Affairs Health Care System.

## Supplementary information

**Additional file 1.** Current State Maps.

**Additional file 2.** Dashboard Questionnaire.

**Additional file 3.** Raw Data.

## Data Availability

All data generated or analysed during this study are included in this published article and its supplementary information files.

## Abbreviations

CBOC: Community-based outpatient clinics
CVT: Clinical Video Telehealth
DMAIC: Define-Measure-Analyze-Improve-Control
eGFR: Estimated glomerular filtration rate
PCP: Primary care provider
PDSA: Plan-Do-Study-Act
SBP: Systolic blood pressure
VA: Veterans Affairs
