# Supplementary material for: Development and implementation of a Telenephrology dashboard for active surveillance of kidney disease: a quality improvement project
Source: BMC Nephrol. 2020 Oct 6;21:424. doi: 10.1186/s12882-020-02077-0 (PMC7539521; doi:10.1186/s12882-020-02077-0)
Supplement: Supplementary file 1 — Additional file 1. Current State Maps. [file 12882_2020_2077_MOESM1_ESM.pdf]

Tele-Nephrology (AKI) – Out of the confidence interval level

Created: 5/1/2018 **Last Updated:** 1/31/2019

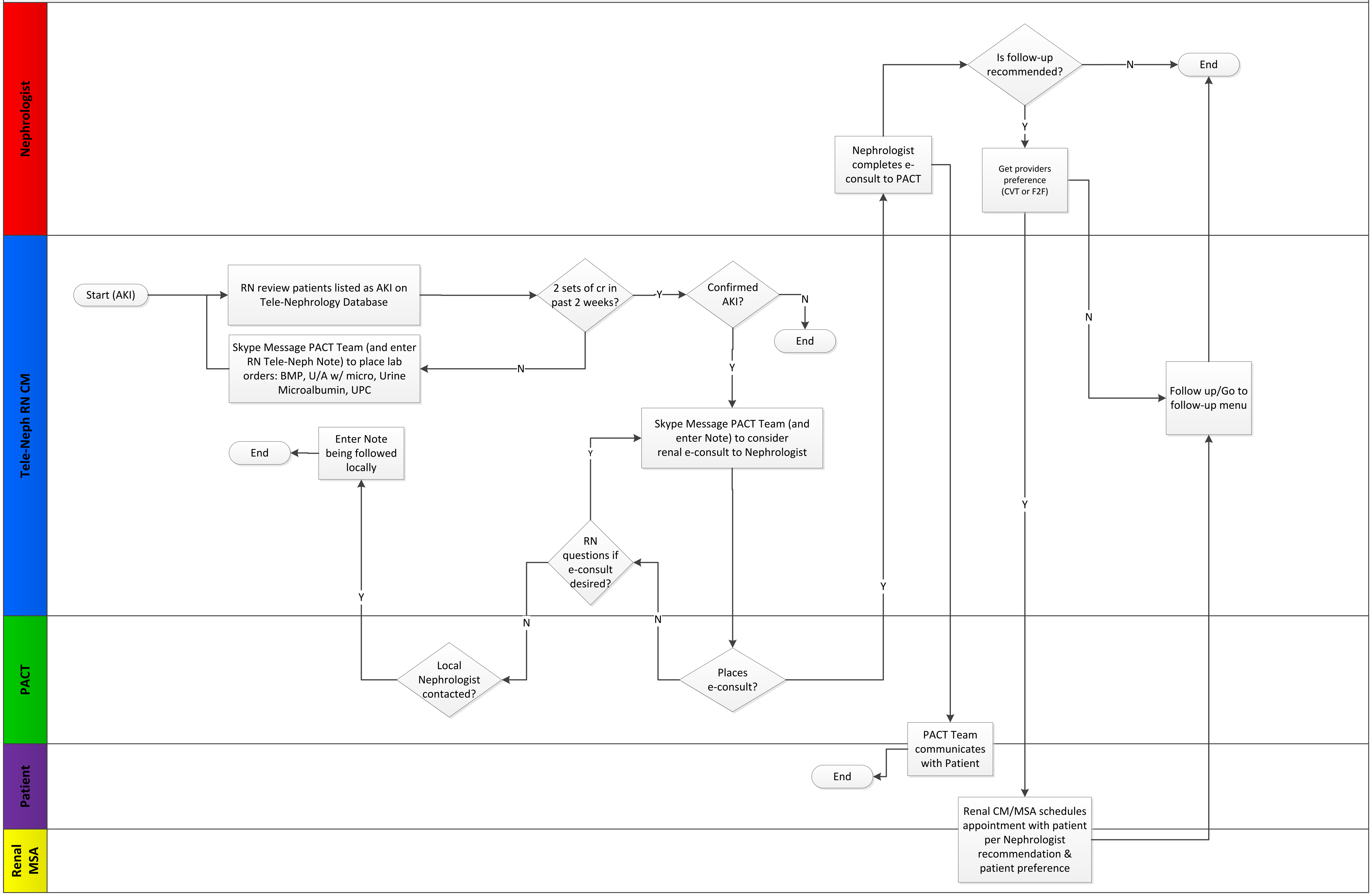

Tele-Nephrology (CKD RISK)

Created: 5/1/2018 **Last Updated:** 1/31/2019

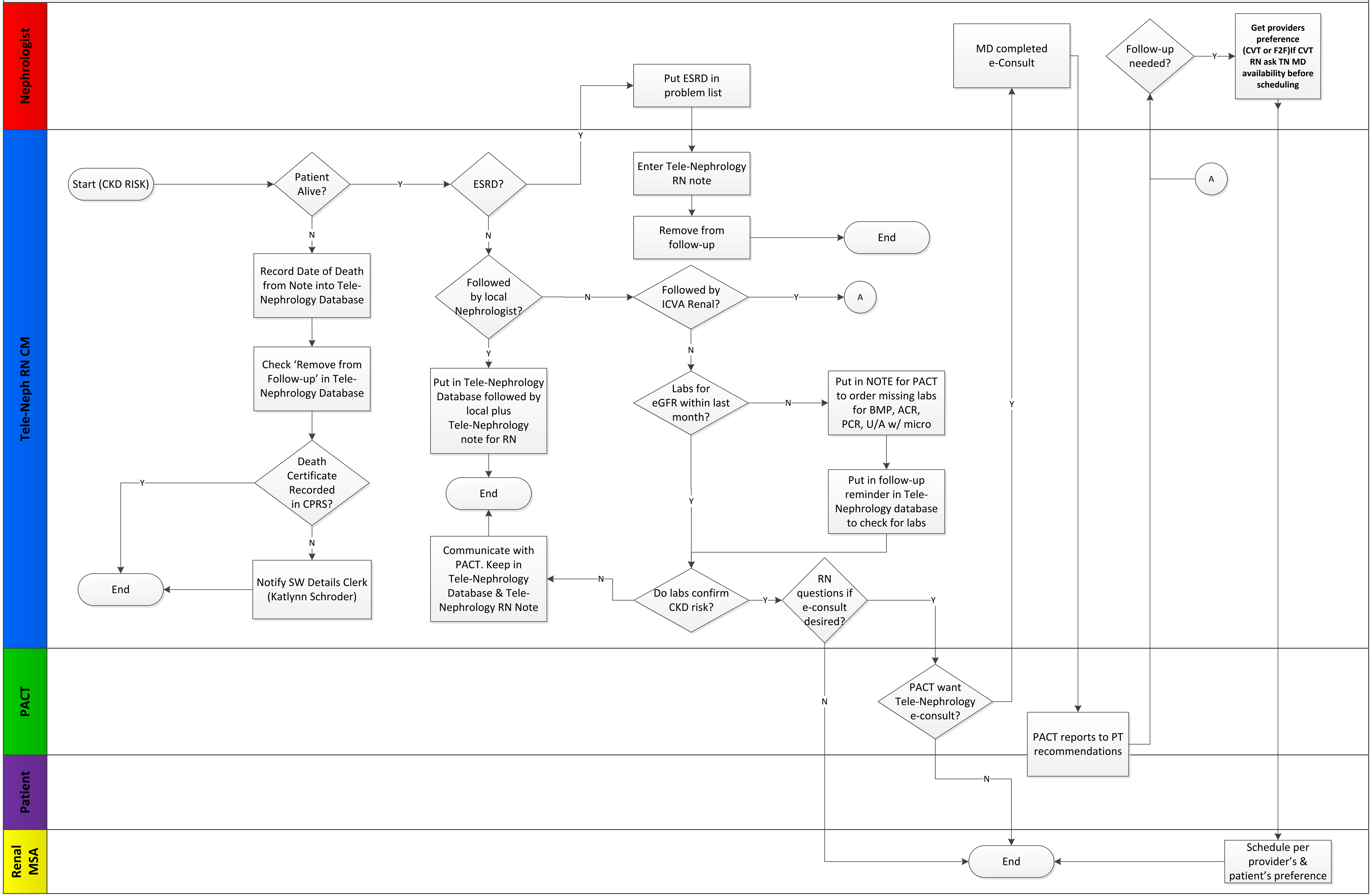

Tele-Nephrology (eGFR)

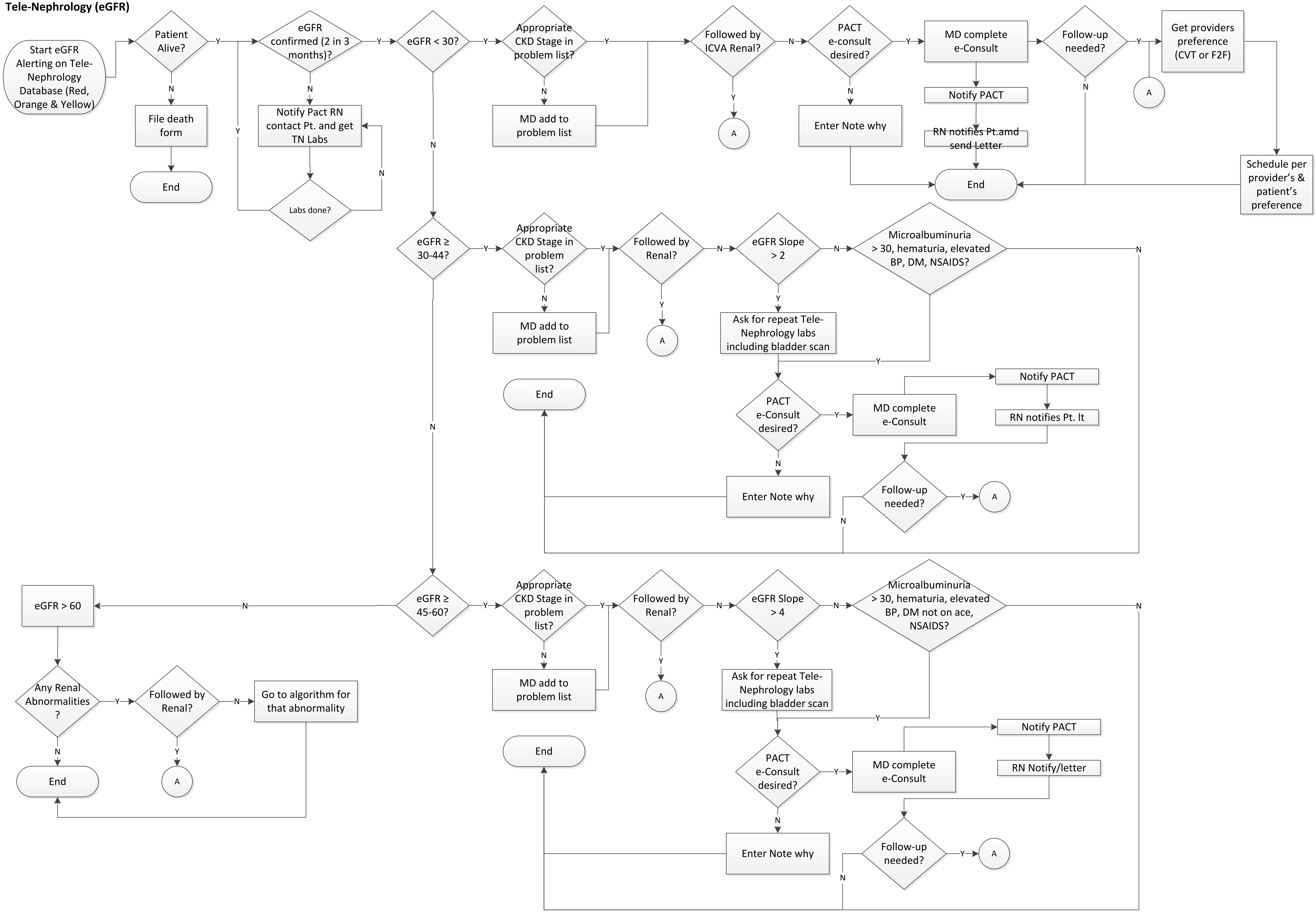

Albuminuria Algorithm

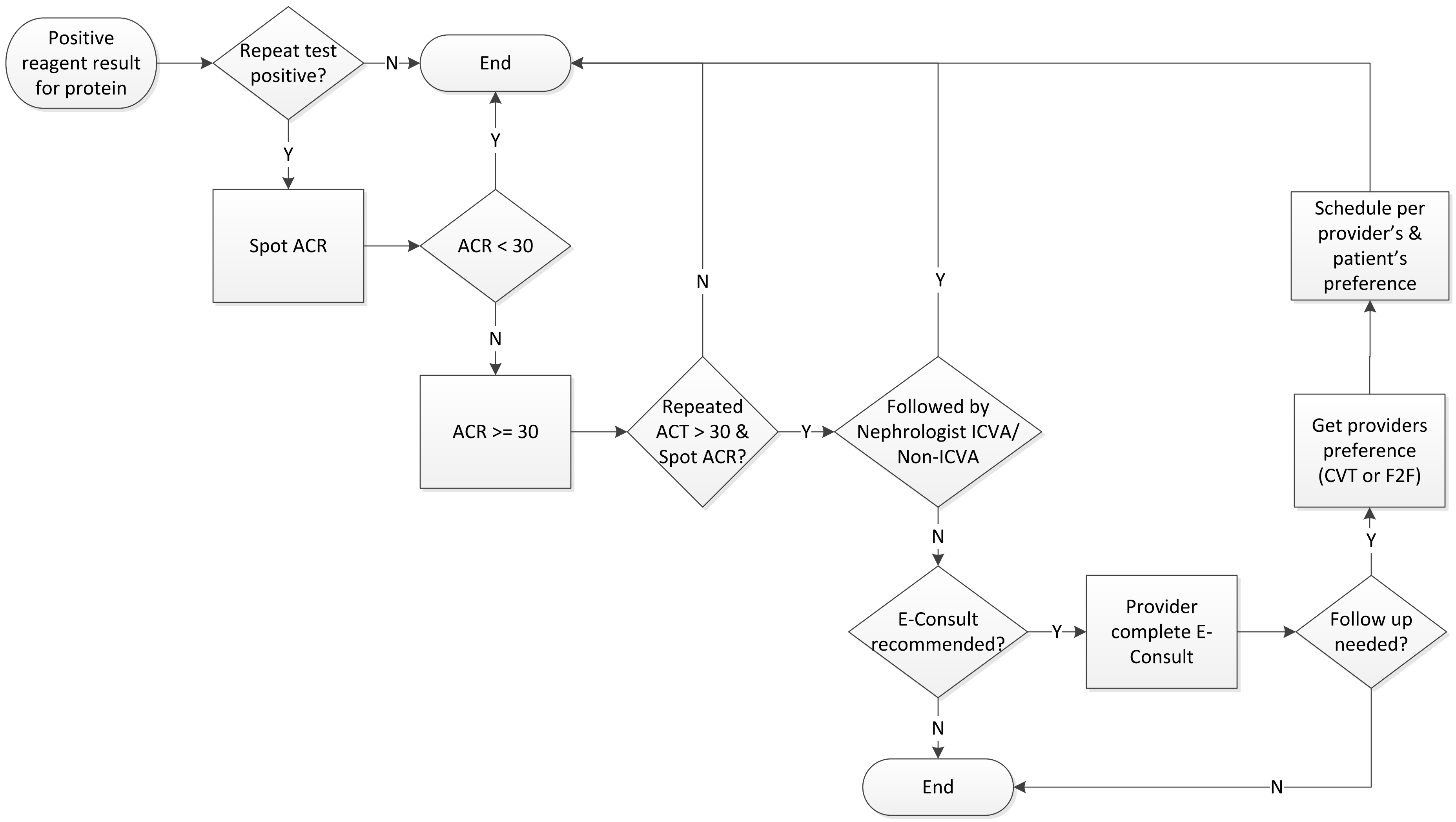

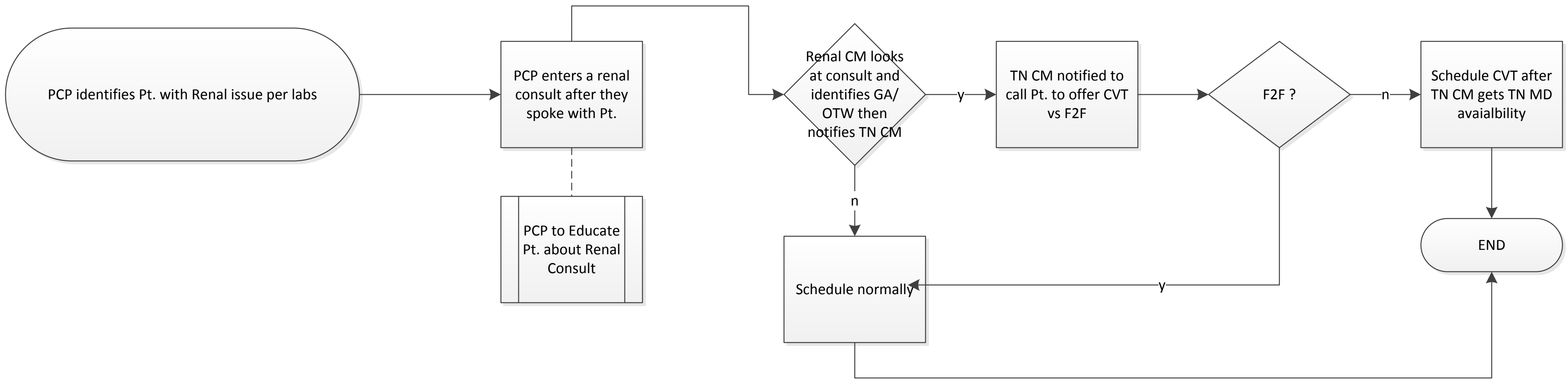
PCP identifies Pt. with Renal issue per labs
PCP enters a renal consult after they spoke with Pt.
PCP to Educate Pt. about Renal Consult
Renal CM looks at consult and identifies GA/ OTW then notifies TN CM
y
n
TN CM notified to call Pt. to offer CVT vs F2F
F2F ?
n
y
Schedule CVT after TN CM gets TN MD avaialbility
Schedule normally
END
