# Supplementary material for: Development and implementation of a Telenephrology dashboard for active surveillance of kidney disease: a quality improvement project
Source: BMC Nephrol. 2020 Oct 6;21:424. doi: 10.1186/s12882-020-02077-0 (PMC7539521; doi:10.1186/s12882-020-02077-0)
Supplement: Supplementary file 2 — Additional file 2. Dashboard Questionnaire. [file 12882_2020_2077_MOESM2_ESM.docx]

For each of the following questions regarding the features of the Dashboard, please answer with one of the options listed below.

# Options:

## I like it that way

## I am expecting it to be that way

## I am neutral

## I can accept it to be that way

## I dislike it that way

# If the Dashboard ***has*** the following feature, how would you feel about it?

|  | A | B | C | D | E |
| --- | --- | --- | --- | --- | --- |
| 1. Ability to drill down to specific events in time |  |  |  |  |  |
| 2. Automatic notifications of lab changes to users |  |  |  |  |  |
| 3. Color markers for indicators |  |  |  |  |  |
| 4. Customization of views based on user |  |  |  |  |  |
| 5. Data tables for displaying serum creatinine, urine blood, and urine protein |  |  |  |  |  |
| 6. Filtering and sorting of values based on user selection |  |  |  |  |  |
| 7. Graphs for kidney function over time |  |  |  |  |  |
| 8. Incorporation of prediction models for development of CKD |  |  |  |  |  |

For each of the following questions regarding the features of the Dashboard, please answer with one of the options listed below.

# Options:

## I like it that way

## I am expecting it to be that way

## I am neutral

## I can accept it to be that way

## I dislike it that way

# If the Dashboard ***does not have*** the following feature, how would you feel about it?

|  | A | B | C | D | E |
| --- | --- | --- | --- | --- | --- |
| 1. Ability to drill down to specific events in time |  |  |  |  |  |
| 2. Automatic notifications of lab changes to users |  |  |  |  |  |
| 3. Color markers for indicators |  |  |  |  |  |
| 4. Customization of views based on user |  |  |  |  |  |
| 5. Data tables for displaying serum creatinine, urine blood, and urine protein |  |  |  |  |  |
| 6. Filtering and sorting of values based on user selection |  |  |  |  |  |
| 7. Graphs for kidney function over time |  |  |  |  |  |
| 8. Incorporation of prediction models for development of CKD |  |  |  |  |  |
